# Biomimetic Salivary Gland Cancer Spheroid Platform for In Vitro Recapitulation of Three-Dimensional Tumor–Stromal Interactions

**DOI:** 10.3390/biom15121634

**Published:** 2025-11-21

**Authors:** Lele Wang, Seokjun Kwon, Sujin Park, Eun Namkoong, Junchul Kim, Hye-Young Sim, Shazid Md. Sharker, Sang-woo Lee

**Affiliations:** 1 Department of Oral Physiology, School of Dentistry and Dental Research Institute, Seoul National University, Seoul 03080, Republic of Korea; wanglele@snu.ac.kr (L.W.); veritas93@snu.ac.kr (S.K.); tnwlsrl@snu.ac.kr (S.P.); eun.namkoong@gmail.com (E.N.); jckim1@snu.ac.kr (J.K.); 2 Department of Dentistry, School of Dentistry and Dental Research Institute, Seoul National University, Seoul 03080, Republic of Korea; ortho72@snu.ac.kr; 3 Department of Dentistry, SMG-SNU Boramae Medical Center, Seoul 07061, Republic of Korea; 4 Department of Pharmaceutical Sciences, North South University, Dhaka 1229, Bangladesh

**Keywords:** salivary gland cancer, cancer-associated fibroblasts, tumor microenvironment, decellularized spheroid, drug screening

## Abstract

Salivary gland carcinomas (SGCs) are aggressive malignancies with limited treatment options, primarily due to the complexity of the tumor microenvironment (TME). Cancer-associated fibroblasts (CAFs) remodel the extracellular matrix (ECM), enhance cancer cell stemness, and drive drug resistance. This study introduces a decellularized CAF-derived spheroid system as a biomimetic platform to study tumor–stromal interactions in SGC. Multicellular spheroids were generated by co-culturing Medical Research Council cell strain 5 (MRC-5) fibroblasts (fetal lung-derived) with A253 salivary gland cancer cells, producing distinct spatial architecture, with fibroblasts at the core and cancer cells at the periphery. Compared with A253-only spheroids, A253/MRC-5 spheroids exhibited enhanced proliferation and elevated expression of stemness markers (aldehyde dehydrogenase 1 [ALDH1], CD133, cytokeratin 19 [CK19]). MRC-5 spheroids displayed robust ECM and growth factor expression that persisted after decellularization. Decellularized spheroids retained biological activity, enabling A253 cells to develop invasive phenotypes, metabolic reprogramming, and stemness-associated signatures. Transcriptomic analysis revealed a transition from proliferative pathways to stress-adaptive survival programs, mirroring in vivo tumor behavior. Moreover, A253 cells cultured with decellularized fibroblast spheroids exhibited altered cisplatin sensitivity, highlighting the critical role of stromal ECM in therapeutic response. In conclusion, this study establishes decellularized CAF spheroids as a simplified yet biologically relevant TME-mimetic platform. By recapitulating tumor–stromal crosstalk without live co-culture, this system provides a powerful tool for mechanistic studies of salivary gland cancer, preclinical drug screening, and development of stroma-targeted therapies.

## 1. Introduction

Salivary gland carcinomas (SGCs) are rare but aggressive malignancies, accounting for approximately 3~6% of all head and neck cancers [1]. Despite their low incidence, they present considerable clinical challenges due to histological diversity, delayed diagnosis, and limited response to conventional therapies [2]. Standard treatments—surgical resection followed by radiotherapy—remain inadequate for advanced or recurrent disease, and systemic chemotherapy (such as cisplatin, paclitaxel, doxorubicin, or their combinations) yields modest benefits [3,4]. The poor prognosis of high-grade subtypes, such as salivary duct carcinoma, underscores the urgent need for improved preclinical models to guide therapeutic development [5]. A significant obstacle in SGC management is the complexity of the tumor microenvironment (TME). Cancer-associated fibroblasts (CAFs), the most abundant stromal component, remodel the extracellular matrix (ECM), secrete growth factors, and establish reciprocal signaling with cancer cells [6]. These interactions sustain tumor growth and invasion while enhancing stemness and promoting drug resistance [7]. Consequently, the TME is increasingly recognized as both a therapeutic barrier and a potential target in SGC therapy [8].

Conventional two-dimensional (2D) cultures fail to reproduce the spatial, biochemical, and mechanical complexity of the TME, limiting their experimental and translational value [9]. In contrast, three-dimensional (3D) systems such as tumor spheroids and organoids reproduce physiologically relevant features-including multicellular architecture, oxygen and nutrient gradients, and cell–matrix interactions, thus improving the fidelity of TME modeling [9]. Co-culture spheroids incorporating stromal populations have further demonstrated that CAFs promote tumor proliferation, ECM remodeling, stemness acquisition, and, in some models, angiogenic signaling and resistance to cytotoxic agents [10,11,12]. However, live stromal co-cultures introduce major analytical limitations. Because fibroblasts remain metabolically and transcriptionally active, their signals mix with those of tumor cells, making it difficult to determine which cell type contributes to outcomes measured by assays such as CCK-8, other viability or metabolic assays, and transcriptomic analyses [13,14,15]. To separate these mixed signals, researchers often need to dissociate spheroids and perform cell-sorting, but these additional steps increase technical complexity and can disrupt the native tumor–stroma organization that 3D models aim to maintain [13,14,15].

To overcome these limitations, a novel SGC culture platform was developed using decellularized Medical Research Council cell strain 5 (MRC-5) CAF spheroids that preserve ECM components, ECM-bound growth factors, and topological characteristics while eliminating cellular interference. This system retains critical biochemical cues of the TME and provides a simplified yet physiologically relevant model. Given the prominence of stromal remodeling and CAF-driven signaling in SGC, decellularized spheroid systems offer strong potential for investigating tumor–stromal interactions, mechanisms of therapeutic resistance, and applications in preclinical drug screening and personalized medicine.

## 2. Materials and Methods

### 2.1. Fabrication of Decellularized Matrices

Human lung fibroblast cells (MRC-5; American Type Culture Collection [ATCC] CCL-171) were cultured in Dulbecco’s Modified Eagle Medium (DMEM) supplemented with 10% fetal bovine serum (FBS) and 1% penicillin–streptomycin (Gibco, Thermo Fisher Scientific, Waltham, MA, USA). Upon reaching confluence, cells were harvested to generate decellularized matrices. For spheroid-derived matrices, MRC-5 spheroids were formed in ultra-low attachment (ULA) U-bottom plates (Corning Inc., Corning, NY, USA; Cat# 7007). Cells were diluted to 1 × 10^7^ cells/mL, and 50,000 cells/well were seeded into ULA culture plates. After 48 h of incubation, spheroids were collected and subjected to decellularization solution treatment for 5 min at room temperature, followed by nuclease treatment for 1 h at 37 °C. All decellularized matrices were thoroughly washed with PBS and stored at 4 °C until further use.

### 2.2. Assembly of A253 and SGT Spheroids

A253 cell line (ATCC HTB-41) derived from human submandibular gland carcinoma [16,17] and Salivary Gland Tumor cell line (SGT-1 cell line) originated from human salivary gland adenocarcinoma cell line [18,19] were kindly donated by professor Kyungpyo Park’s lab in Department of Oral Physiology, Seoul National University School of Dentistry. Cells were cultured in DMEM supplemented with 10% FBS and 1% penicillin–streptomycin. Upon confluence, cells were harvested and seeded at 8000 cells/well on ULA plates (Corning Inc.; Cat# 7007), with or without decellularized matrices. Spheroid clusters were harvested on Day 3. Cell viability was assessed using the Cell Counting Kit-8 (CCK-8; Dojindo, Kumamoto, Japan).

### 2.3. Imaging

Bright-field images of spheroids were acquired using an inverted microscope (Nikon, Tokyo, Japan). Fluorescence images were captured using a confocal laser scanning microscope (CLSM; LSM 980, Zeiss, Jena, Germany) operated via ZEN 3.6 Blue software.

### 2.4. Dual-Color Labeling of Co-Culture Spheroids

MRC-5 fibroblasts and A253 SGC cells were pre-labeled with CellTracker Red and Green (Thermo Fisher Scientific; Cat# C34565 and C2925) by incubation with 2 μM dye for 30 min at 37 °C. After washing with PBS, cells were seeded into ULA plates to form spheroids. After 48 h, co-culture spheroids were imaged using CLSM.

### 2.5. Immunofluorescence Staining

A253 cells cultured with decellularized matrices were fixed in 4% paraformaldehyde for 20 min, subsequently blocked and permeabilized in PBS containing 0.1% Triton X-100 and 10% normal donkey serum (NDS; Sigma-Aldrich, St. Louis, MO, USA; Cat# D9663) for 3 h at room temperature. Samples were incubated overnight at 4 °C with anti-E-Cadherin primary antibody (Abcam, Cambridge, UK; ab40772, rabbit monoclonal, 1:100), followed by Donkey Anti-Rabbit IgG H&L Alexa Fluor^®^ 488 secondary antibody (Invitrogen, Thermo Fisher Scientific; Cat# R37118, 1:250) for 1 h. Nuclei were counterstained with 4′,6-diamidino-2-phenylindole (DAPI; 1.5 μg/mL, Santa Cruz Biotechnology, Dallas, TX, USA) and mounted in Fluorogel (ProSciTech, Townsville, Australia; Cat# IM030). Imaging was performed using CLSM (LSM 700, ZEISS, Oberkochen, Germany).

### 2.6. Nucleic Acid and Protein Extraction

For protein extraction, MRC-5 spheroids and decellularized spheroids were lysed on ice in Radioimmunoprecipitation Assay (RIPA) buffer (1 mL per 107 cells), incubated for 30 min, homogenized by pipetting, and centrifuged at 14,000× *g* for 15 min at 4 °C. Supernatants were collected for protein profiling. For nucleic acids, RNA and DNA were extracted using the ZymoBIOMICS DNA/RNA Kit (Zymo Research, Irvine, CA, USA; Cat# R2002). RNA from A253 cells and A253 cells co-cultured with decellularized spheroids was used for quantitative real-time polymerase chain reaction (qRT-PCR) and transcriptome sequencing. DNA from MRC-5 spheroids and decellularized spheroids was analyzed to confirm decellularization efficiency.

### 2.7. Quantitative Real-Time RT-PCR (qRT-PCR)

Total RNA was reverse-transcribed to complementary DNA (cDNA) using the PrimeScript-based kit (Takara Bio, Shiga, Japan; Cat# RR036A). The resulting cDNA was diluted 1:10 in nuclease-free water for qRT-PCR using SYBR Green (Takara Bio; Cat# RR820A). Gene-specific primers were as follows: aldehyde dehydrogenase 1 (ALDH1) (Forward: 5′-CGTGGCGTACTATGGATGCT-3′, Reverse: 5′-CTTTCCTCCAAGCTCCAGGG-3′), CD133 (Forward: 5′-TGTGCGGGAACTCCTTTCA-3′, Reverse: 5′-GTAGCATGTGGTACAGCCCA-3′), cytokeratin 19 (CK19) (Forward: 5′-AGATAACGCTGTGCTGCAGT-3′, Reverse: 5′-TCCACATTGACTGTCCAGCC-3′), and glyceraldehyde-3-phosphate dehydrogenase (GAPDH) (Forward: 5′-GCTCCCTCTTTCTTTGCAGC-3′, Reverse: 5′-ACCATGAGTCCTTCCACGAT-3′). The Ct values were normalized to GAPDH, and relative expression was determined using the ΔCt method. Primer specificity was confirmed by melt-curve analysis.

### 2.8. Morphological Characterization of Decellularized Matrices

Field emission scanning electron microscopy (FE-SEM) was used to examine decellularized matrices. Samples were fixed with 3.7% paraformaldehyde for 4 h, dehydrated in graded ethanol (30%, 50%, 70%, 100%), and dried with a critical point dryer (Tousimi, Rockville, MD, USA). Samples were coated with 6–8 nm gold (Emitech K575) and imaged using an Apreo S FE-SEM (Thermo Fisher Scientific) operated at 5–10 kV with a working distance of 4–8 mm.

### 2.9. Calcein AM/Propidium Iodide (PI) Co-Staining of Spheroids

To evaluate growth and cell viability, A253 and SGT spheroids cultured with decellularized MRC-5 spheroid-derived ECM or control conditions were stained with Calcein AM and propidium iodide (PI). Spheroids were incubated in a culture medium containing Calcein AM (2 μM; Invitrogen, Waltham, MA, USA; Cat# C3100MP) and PI (2 μg/mL; Sigma-Aldrich; Cat# P4864) for 30 min at 37 °C, then washed twice with PBS. Images were acquired using CLSM (LSM 700, ZEISS, Oberkochen, Germany) and inverted microscope (Nikon, Tokyo, Japan).

### 2.10. Liquid Chromatography-Mass Spectrometry (LC-MS) Analysis of Decellularized Matrices

Protein concentration was quantified using the Pierce BCA Protein Assay Kit (Thermo Fisher Scientific). Samples were digested using Filter-Aided Sample Preparation (FASP) on Microcon 30K centrifugal filters (Millipore, Billerica, MA, USA). Reduction was performed with Tris(2-carboxyethyl)phosphine (TCEP) at 37 °C for 30 min, followed by alkylation with iodoacetic acid (IAA) at 25 °C for an hour in the dark. After sequential washing with lysis buffer and 50 mM ammonium bicarbonate (ABC), proteins were digested with trypsin (enzyme: protein ratio of 1:50; *w*/*w*) at 37 °C for 18 h. Digested peptides were transferred to new tubes, inactivated with 15 μL of formic acid (Honeywell, Charlotte, NC, USA), desalted using C18 spin columns (Harvard Apparatus, Holliston, MA, USA), and eluted with 80% acetonitrile in 0.1% formic acid. Peptides were resuspended in 0.1% formic acid and analyzed using a Q-Exactive Orbitrap hybrid mass spectrometer (Thermo Fisher Scientific, Waltham, MA, USA) with an Ultimate 3000 system (Thermo Fisher Scientific, Waltham, MA, USA). Separation was achieved with a 2 cm × 75 μm ID trap column packed with 3 μm C18 resin and a 50 cm × 75 μm ID analytical column packed with 2 μm C18 resin. The mobile phases were (A) 0.1% formic acid in water and (B) 0.1% formic acid in 90% acetonitrile, at 300 nL/min. The gradient was: 4% solvent B in (0–14 min), 4–15% B (15–76 min), 15–28% B (77–127 min), 28–40% B (128–147 min), 40–96% B (148–150 min), hold at 96% B (151–163 min), 96–4% B (164 min), and re-equilibrate at 4% B (165–189 min). Data-dependent acquisition selected the top 10 precursor peaks for fragmentation. MS1 scans had a resolution of 70,000, and MS2 had 17,500 (*m*/*z* 400), which was over a range of 400–2000 *m*/*z*. Precursor ions were fragmented at normalized collisional energy (NCE) 27%, with a dynamic exclusion of 30 s.

### 2.11. Transcriptome Sequencing and Functional Analysis

Sequencing was performed on an Illumina NextSeq 500 platform (Illumina, San Diego, CA, USA) to generate 50 bp single-end reads. Library preparation was outsourced to Ebiogen, Inc. (Seoul, Republic of Korea) using the QuantSeq 3’ mRNA-Seq Library Prep Kit (Lexogen, Greenland, NH, USA). Raw reads were processed and normalized with the edge R package v4 in R using quantile normalization. Differentially expressed genes (DEGs) were defined as those with *p* < 0.05, normalized expression ≥ 4, and absolute fold change ≥ 2. Functional enrichment was performed using DAVID Bioinformatics Resources 6.8 (https://davidbioinformatics.nih.gov/home.jsp (accessed on 13 September 2025)), including Gene Ontology (GO) and Kyoto Encyclopedia of Genes and Genomes (KEGG) analyses. Enriched GO terms included extracellular matrix structural constituent (GO:0005201) and growth factor activity (GO:0008083). Pathways or terms with *p* < 0.05 were considered significant.

### 2.12. Metabolic Activity Using 2-(N-(7-Nitrobenz-2-oxa-1,3-diazol-4-yl) Amino)-2-Deoxyglucose (2-NBDG) Uptake Assay

Glucose uptake was assessed using the fluorescent analog 2-NBDG. A253 spheroids cultured in ULA wells, with or without decellularized MRC-5 spheroids, were incubated in glucose-free DMEM for 60 min and then exposed to 1 mM 2-NBDG for 30 min. Cells were counterstained with Hoechst 33342. Fluorescence (Ex/Em: 475/550 nm for 2-NBDG, 350/461 nm for Hoechst) was visualized with a CLSM (LSM 700, ZEISS, Oberkochen, Germany). Data were analyzed using ZEN 3.6 Blue software.

### 2.13. Caspase-3/7 Assay for Anticancer Activity

A253 spheroids cultured with or without decellularized MRC-5 scaffolds were treated with cisplatin (7, 15, or 30 μM; Sigma-Aldrich), doxorubicin (1, 5, or 10 μM; Sigma-Aldrich), and paclitaxel (0.1, 1, or 10 μM; Sigma-Aldrich). After 24 h of treatment, apoptotic activity was assessed using the CellEventTM caspase-3/7 green detection reagent (2 μM; Invitrogen, ThermoFisher Scientific, Cat# C10423). The reagent was added and incubated for approximately 2 h at 37 °C. Live-cell fluorescence images were subsequently acquired using a Leica digital inverted fluorescence microscope.

### 2.14. Pharmacologic Inhibition of FAK and EGFR Signaling

To evaluate the roles of ECM–FAK and EGFR signaling in ECM-induced stemness, A/M (DC) spheroids were treated with a FAK inhibitor (Sigma-Aldrich; Cat# SML0837; 5 µM, 24 h) or Erlotinib (Sigma-Aldrich; Cat# SML2156; 20 µM, 24 h) under identical culture conditions. Following treatment, total RNA was isolated for quantitative RT-PCR analysis of stemness-associated genes.

### 2.15. Statistical Analysis

Quantitative data are expressed as mean ± standard deviation (SD). Statistical significance was determined using an unpaired *t*-test or one-way analysis of variance (ANOVA) with Tukey’s post hoc test (GraphPad Prism 10). A *p*-value < 0.05 was considered statistically significant.

## 3. Results and Discussion

### 3.1. Spatial Organization of A253/MRC-5 Co-Culture Spheroids and Its Impact on Stemness

**Figure 1:**
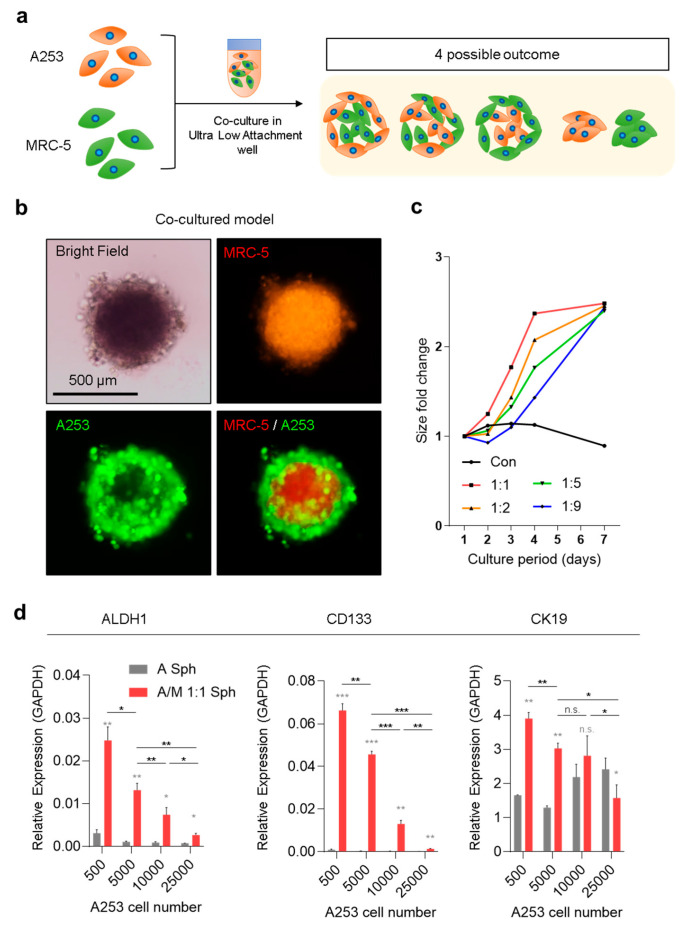
Geometric and molecular patterns of A253/MRC-5 co-culture model. (**a**) Schematic illustration of the co-culture between cancer-associated fibroblast (MRC-5) cells and A253 salivary gland carcinoma cells, highlighting four potential interaction outcomes. (**b**) Epifluorescence images of MRC-5 fibroblasts (Cell Tracker Red-labeled)/A253 (Cell Tracker Green-labeled) with co-culture spheroids. (**c**) Fold change in spheroid size at different MRC-5/A253 ratios (1:1, 1:2, 1:5, 1:9) during 1–7 days of culture. (**d**) qRT-PCR analysis of tumor stem cell markers ALDH1, CD133, and CK19, normalized to GAPDH. Data represent mean ± standard deviation (SD) from three biological replicates. Statistical analysis was conducted using one-way ANOVA and an unpaired *t*-test. n.s., not significant, * *p* < 0.05, ** *p* < 0.01, *** *p* < 0.001.

The co-culture system of MRC-5 and A253 SGC cells was designed to examine potential spatial arrangements arising from stromal–tumor interactions (Figure 1a). Four geometric outcomes were considered: (i) MRC-5 cells at the periphery with A253 cells in the core, (ii) homogeneous mixing of both cell types, (iii) MRC-5 cells forming the core with A253 cells at the periphery, and (iv) physically separated, non-interacting clusters. Among these, spheroids exhibited distinct compartmentalization, with MRC-5 fibroblasts localized to the inner core and A253 cells forming the outer layer (Figure 1b). This spatial separation aligns with the stromal function of fibroblasts, suggesting that MRC-5 cells create a structural niche that supports carcinoma growth. Proliferation, measured as fold change in spheroid size, increased across all tested MRC-5:A253 ratios, with maximal growth observed at a 1:1 ratio (Figure 1c). At this ratio, spheroid size plateaued by Day 4, identified as the optimal time point for generating complex spheroids in subsequent experiments (Figure 1c). This 1:1 (MRC-5:A253) co-culture condition also showed elevated expression of cancer stemness markers—ALDH1, CD133, and CK19—compared with A253-only spheroids (Figure 1d). These findings highlight the synergistic role of fibroblasts in promoting stem cell–like properties in carcinoma cells. Interestingly, expression of stemness markers declined as the initial cell number increased, despite overall spheroid enlargement (Figure 1d). This reduction likely reflects a lower surface-to-volume ratio in larger spheroids, restricting direct stromal–tumor cell interactions. Such contact-dependent signaling appears critical for sustaining stem-like phenotypes, consistent with previous reports that CAF-mediated cell-to-cell interactions drive tumor aggressiveness [20].

### 3.2. Global Transcriptomic Analysis of MRC-5 Spheroids

**Figure 2:**
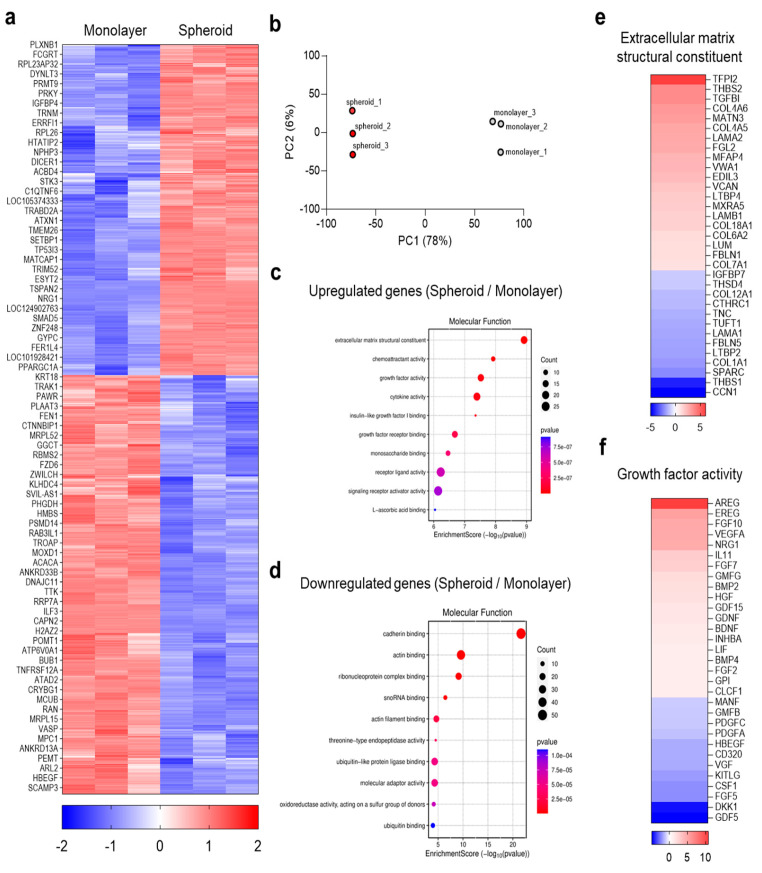
Transcriptomic profiling of MRC-5 fibroblasts in 3D spheroids. (**a**) Heatmap of differentially expressed genes (DEGs) comparing monolayer- versus spheroid-cultured MRC-5 (n = 3). (**b**) Principal component analysis (PCA) of MRC-5 cultured as spheroids and monolayers (PC1~78%). (**c**) Gene Ontology (GO) analysis of “Molecular Function” enrichment among upregulated genes (Fold change ≥ 2, Normalized counts ≥ 4, *p* < 0.05). (**d**) GO analysis with “Molecular Function” enrichment among downregulated genes (Fold change ≥ 2, Volume ≥ 4, *p* < 0.05). (**e**) DEGs categorized under “Extracellular matrix structural constituent” (GO:0005201; Fold change ≥ 2, Volume ≥ 4, *p* < 0.05). (**f**) DEGs categorized under “Growth factor activity” (GO:0008083; Fold change ≥ 2, Volume ≥ 4, *p* < 0.05.

The spatial compartmentalization observed in the A253/MRC-5 co-culture system underscored the pivotal role of MRC-5 spheroids, positioned at the spheroid core, in reprogramming tumor growth and stemness. To investigate the molecular basis of this phenomenon, mRNA sequencing was performed to compare transcriptomes of MRC-5 fibroblasts cultured as monolayers versus spheroids. Spheroid culture was hypothesized to induce a CAF-like transcriptional program with elevated ECM and growth factor gene expression. Global transcriptomic profiling confirmed this hypothesis, revealing a clear segregation between monolayer- and spheroid-cultured fibroblasts (Figure 2a,b). Hierarchical clustering demonstrated extensive transcriptomic reprogramming, and PCA distinctly separated the groups, indicating a robust culture-mode effect (Figure 2a,b). Functional enrichment analyses showed that spheroid-upregulated genes were significantly associated with ECM structure, growth factor activity, cytokine/receptor signaling, and insulin-like growth factor (IGF)-binding functions (Figure 2c). Conversely, downregulated pathways involved ribonucleoprotein/translation processes and cytoskeletal or cadherin-binding activities (Figure 2d), consistent with a shift from proliferative housekeeping toward a secretory, matrix-remodeling phenotype typical of activated CAFs [21]. The ECM-associated heatmap further highlighted enhanced expression of fibrillar and matricellular proteins, including COL1/6/12, FN1, laminins, tenascin-C, SPARC, thrombospondins, TGFBI, and LTBPs (Figure 2e). Such remodeling enhances ligand availability and mechanotransduction via integrin β1/FAK-PI3K/AKT and ERK signaling, thereby promoting carcinoma proliferation, invasion, and stemness [22,23]. Fibrillar collagens and tenascin-C, in particular, are directly linked to cancer stem cell (CSC) marker enrichment and therapeutic resistance [23,24]. Parallel “growth factor activity” analysis revealed strong upregulation of paracrine mediators, including AREG/EGFR ligands, FGFs, VEGFA/PGF, NRG1 (ERBB ligand), HGF (MET ligand), BMP/GDF family members, PDGFs, and IGF-axis/IGFBPs (Figure 2f). CAF-derived amphiregulin and neuregulins are known activators of ERK/AKT signaling, increasing epithelial cancer cell survival, plasticity, and therapy tolerance through reciprocal stromal communication [25,26,27]. Similarly, HGF-MET and FGF-FGFR pathways are established drivers of proliferation, invasion, and CSC maintenance in head and neck tumors [28,29,30]. Together, these findings demonstrate that MRC-5 fibroblasts in 3D spheroid culture adopt a CAF-like transcriptional program defined by ECM remodeling and growth factor secretion. These stromal cues provide both contact-dependent and paracrine inputs, explaining the enhanced A253 proliferation and stemness observed in co-culture. Therefore, MRC-5 spheroids represent a physiologically relevant CAF model and a robust platform for dissecting stromal regulation of tumor progression and therapy response. While MRC-5 fibroblasts were used as the stromal source in this study, it is acknowledged that these cells are fetal lung-derived and not patient-derived CAFs. Nevertheless, the transcriptomic data indicate that MRC-5 spheroids acquire a CAF-like profile characterized by ECM remodeling and elevated growth-factor signaling (Figure 2), supporting their biological suitability as a stromal mimic. MRC-5 was selected because there is currently no salivary-gland-specific CAF cell line available, and its GMP-certified, well-characterized properties ensure reproducibility and consistency for mechanistic studies. Future work will aim to apply this decellularized-spheroid strategy to patient-derived CAFs to evaluate inter-patient variability and further validate the platform.

### 3.3. Generation and Characterization of Decellularized MRC-5 Spheroids

**Figure 3:**
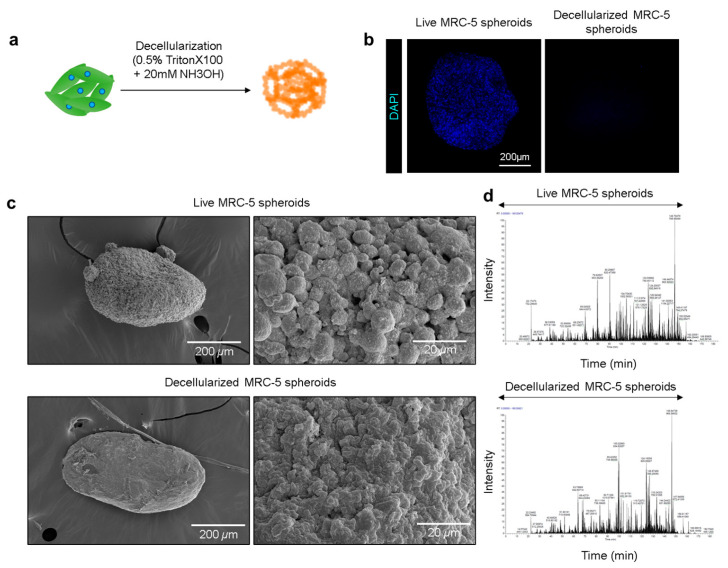
Generation and characterization of decellularized MRC-5 spheroid scaffolds. (**a**) Schematic illustration of the procedure for preparing decellularized MRC-5–derived matrices by incubation in a water–surfactant mixture. (**b**) DAPI staining of live MRC-5 spheroids versus decellularized spheroid matrices. (**c**) SEM images of live and decellularized MRC-5 spheroids with corresponding magnified views. Scale bars: 200 µm (left) and 20 µm (right). (**d**) LC-MS full-scan chromatograms of live spheroids (top) and decellularized spheroid matrices (bottom) (See Figure S1 for details).

Based on our observation that MRC-5 fibroblasts positioned at the core of A253/MRC-5 co-culture spheroids enhanced A253 proliferation and stemness through enriched ECM molecules and growth factor secretion, we sought to establish a simplified stromal scaffold that preserved these tumor-supportive cues while eliminating live-cell signals that could confound downstream analyses. To this end, decellularized MRC-5 spheroids were generated as stromal mimics designed to retain the structural and biochemical features of the TME while eliminating fibroblast transcriptional activity. MRC-5 spheroids were first established using ULA plates and subjected to detergent-based decellularization (0.5% Triton X-100 with 20 mM ammonium hydroxide [NH_4_OH]) (Figure 3a). This treatment effectively lysed the cells and removed nuclear material, leaving an ECM-rich scaffold. DAPI staining confirmed abundant nuclear signals in live spheroids, whereas none were detected in decellularized spheroids, verifying effective cellular removal (Figure 3b). Structural integrity was then assessed using SEM. Live spheroids exhibited a granular, porous surface consistent with dense cell–cell and cell–matrix interactions (Figure 3c, upper panel). Conversely, decellularized spheroids retained overall three-dimensional architecture but displayed a smoother, amorphous morphology with scattered particulate features, consistent with ECM exposure following cell removal (Figure 3c, lower panel). To confirm ECM preservation, LC-MS full-scan analysis was performed. Chromatographic profiles revealed that most ECM-associated molecular species were detectable in both live and decellularized spheroids within the 40–150 min retention range (Figure 3d and Figure S1). Overall peak distribution patterns were highly similar, indicating conservation of key ECM constituents following decellularization (Figure 3d and Figure S1). Some ECM-associated peaks were even more abundant in decellularized samples, suggesting enrichment of structural proteins after removal of intracellular content. Collectively, these results demonstrate that decellularized MRC-5 spheroids yield ECM-rich, structurally preserved scaffolds that recapitulate the CAF-derived stromal microenvironments, providing a biologically relevant platform for modeling salivary gland tumor–stroma interactions.

### 3.4. Physiological Characterization of A253 Cells Cultured with Decellularized MRC-5 Spheroid Scaffolds

**Figure 4:**
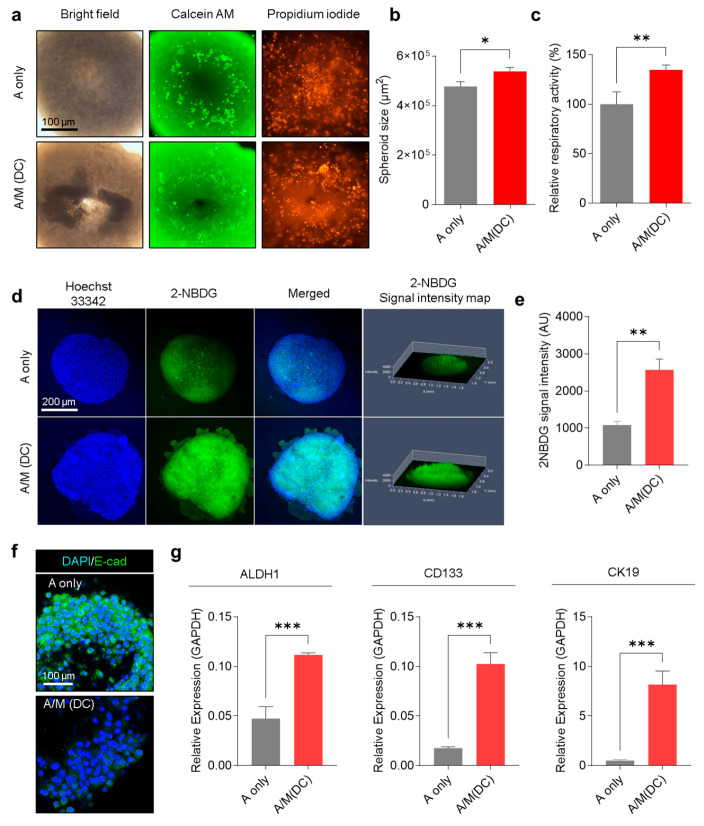
Physiological characterization of A253 cells cultured with or without decellularized MRC-5 scaffolds. (**a**) Bright-field and Calcein AM/PI co-staining of A only vs. A/M(DC) spheroids. Representative images of four replicates. (**b**) Spheroid cross-sectional area (µm^2^) in A-only vs. A/M(DC) spheroids (n = 3). (**c**) Respiratory activity (%) of A only vs. A/M(DC) spheroids measured by CCK-8 assay (n = 3). (**d**) Confocal microscope images of A only and A/M(DC) spheroids stained with Hoechst 33342 (blue) and 2-NBDG (green). Representative images of three replicates. (**e**) Quantification of 2-NBDG intensity (n = 3). (**f**) Confocal microscopic images of A only and A/M(DC) spheroids immunostained for E-cadherin (green) and DAPI (blue). Representative images of three replicates. (**g**) mRNA expression of ALDH1, CD133, and CK19 in A-only vs. A/M(DC) spheroids (n = 3). Data shown as mean ± standard deviation (±SD). Statistical analysis conducted using an unpaired *t*-test. * *p* < 0.05, ** *p* < 0.01, *** *p* < 0.001.

Having shown that decellularized MRC-5 spheroids preserve ECM integrity and retain bioactive components, their functional effects on SGC cells were evaluated. A253 spheroids cultured with decellularized MRC-5 spheroids (A/M(DC)) displayed altered growth dynamics and metabolic activity compared with A253-only spheroids (A only). Bright-field imaging and Calcein AM/PI staining revealed larger spheroid size and higher viability in A/M(DC) cultures (Figure 4a). Quantitative analysis confirmed a substantial 12% increase in spheroid diameter (Figure 4b). To confirm that these ECM-mediated effects were not limited to A253 cells, additional experiments were performed using the human salivary gland tumor (SGT) cell line under identical conditions. SGT/M (DC) spheroids exhibited larger spheroid size and fewer PI-positive cells compared with SGT-only spheroids (Figure S3a,b), demonstrating consistent ECM-dependent enhancement of growth and viability across salivary gland tumor models. Respiratory activity was elevated by 34.6% relative to A-only spheroids, suggesting enhanced metabolic activity (Figure 4c). To further investigate this metabolic shift, glucose uptake was assessed using the fluorescent tracer 2-NBDG. Confocal imaging demonstrated more substantial intracellular 2-NBDG accumulation in A/M(DC) spheroids, with quantitative analysis confirming significantly higher uptake compared to A-only controls (Figure 4d,e). Beyond proliferation and metabolism, A/M(DC) scaffolds also influenced A253 phenotype. Immunofluorescence staining revealed considerable downregulation of E-cadherin (Figure 4f and Figure S4), indicating epithelial–mesenchymal transition (EMT) and acquisition of invasive, mesenchymal-like traits [31]. The qRT-PCR analysis demonstrated significant upregulation of stemness-associated markers (ALDH1, CD133, and CK19) in A/M(DC) spheroids compared with A-only spheroids (Figure 4g). Consistently, SGT/M (DC) spheroids showed elevated expression of ALDH1, CD133, and CK19 relative to SGT-only spheroids (Figure S3c). This enrichment of stem-like markers underscores the ability of fibroblast-derived ECM to promote aggressive tumor phenotypes. These findings demonstrate that decellularized MRC-5 spheroid-derived ECM functions as a biologically active scaffold that maintains structural and biochemical properties, and recapitulates stromal influences on tumor progression. By promoting proliferation, metabolic activation, EMT induction, and cancer stemness, these scaffolds effectively reproduce the tumor-supportive role of CAFs within a simplified and controllable experimental platform.

### 3.5. Evaluation of Chemotherapeutic Resistance in A253 Spheroids Cultured with Decellularized MRC-5 Spheroid Scaffolds

**Figure 5:**
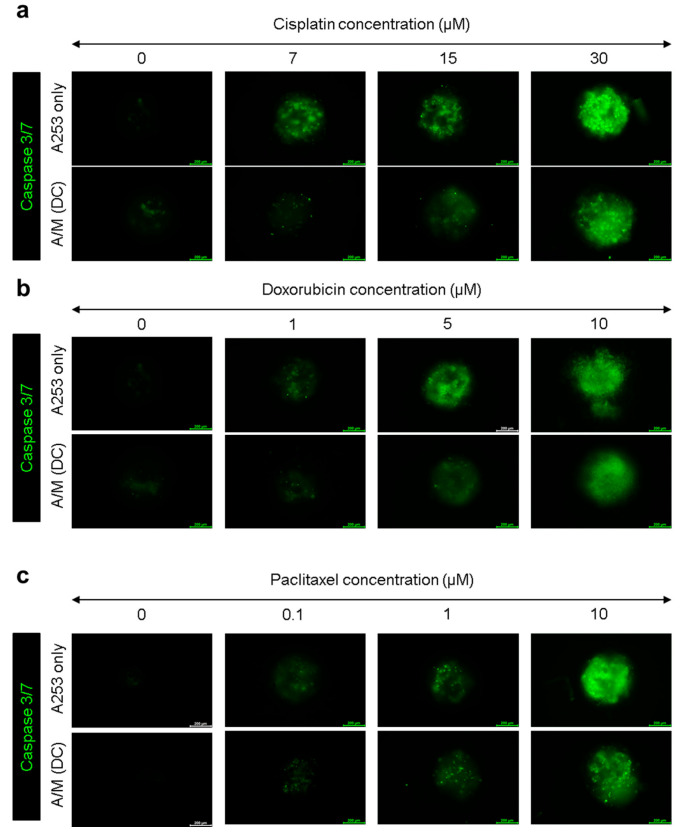
Evaluation of multidrug resistance in A253 spheroids cultured with or without decellularized MRC-5 scaffolds. (**a**) Caspase-3/7 fluorescence images of A253-only and A/M (DC) spheroids treated with cisplatin (0, 7, 15, 30 µM). (**b**) Caspase-3/7 fluorescence images of A253-only and A/M (DC) spheroids treated with doxorubicin (0, 1, 5, 10 µM). (**c**) Caspase-3/7 fluorescence images of A253-only and A/M (DC) spheroids treated with paclitaxel (0, 0.1, 1, 10 µM). All images show representative fluorescence from three biological replicates. Scale bars: 200 µm.

To evaluate whether decellularized MRC-5 spheroid scaffolds modulate the chemotherapeutic response of salivary-gland tumor cells, A253 spheroids cultured with or without scaffolds were treated with cisplatin, doxorubicin, or paclitaxel at various concentrations for 24 h. Caspase-3/7 fluorescence imaging revealed clear differences in apoptotic activation between groups (Figure 5). In A253-only spheroids, all drugs induced dose-dependent increases in caspase-3/7 signal, indicating progressive apoptosis. In contrast, A/M (DC) spheroids exhibited markedly weaker fluorescence across all treatments, demonstrating that the decellularized fibroblast matrix confers broad resistance to cytotoxic stress.

These data support ECM-mediated drug resistance, wherein integrin/FAK–PI3K/AKT and ERK signaling downstream of collagen-, laminin-, tenascin-C-rich matrices (upregulated in MRC-5 spheroids) suppress apoptosis and promote survival under cytotoxic stress [32,33]. In parallel, the paracrine ligand milieu (such as AREG/EGFR, HGF/MET, FGFs) together with EMT and CSC enrichment observed in our model are established mediators of platinum tolerance in epithelial tumors [34,35,36]. To further verify the involvement of the integrin–FAK axis, A/M (DC) spheroids were treated with a FAK inhibitor for 24 h. Expression of stemness markers ALDH1, CD133, and CK19 decreased compared with untreated A/M (DC) spheroids (Figure S4), confirming that ECM–FAK signaling contributes to stemness and survival under chemotherapeutic stress. Together, these findings demonstrate that ECM-driven activation of the FAK and related pathways underlies the resistance of A/M (DC) spheroids to cisplatin, doxorubicin, and paclitaxel, and reinforce the value of this stromal-mimetic platform for preclinical drug testing in SGC. However, it remains necessary to validate in vivo-using an appropriate xenograft model to assess whether the phenotypic changes observed in salivary gland cancer cells cultured with decellularized MRC-5 spheroids can be recapitulated under physiological conditions.

### 3.6. Transcriptomic Reprogramming of A253 Spheroids Cultured with Decellularized MRC-5 Spheroid Scaffolds

**Figure 6:**
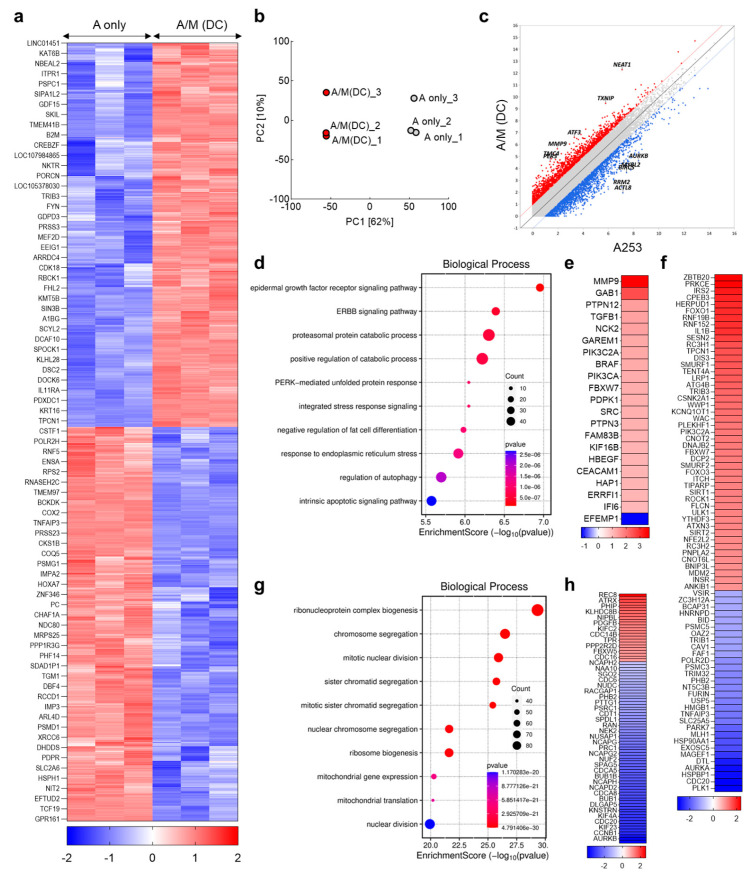
Transcriptomic reprogramming of A253 spheroids by decellularized MRC-5 spheroid scaffolds. (**a**) Heatmap of DEGs (A only vs. A/M(DC)); rows scaled by z-score (n = 3). (**b**) Principal component analysis (PCA) of A only and A/M(DC) groups. (**c**) Scatter/MA plot highlighting top up- and down-regulated genes in A/M(DC). (**d**) Gene Ontology (GO) analysis with “Molecular Function” enrichment among downregulated genes (Fold change ≥ 2, Volume ≥ 4, *p* < 0.05). (**e**) Differentially expressed genes categorized under “Epidermal growth factor receptor signaling pathway” (GO:0007173; Fold change ≥ 2, Volume ≥ 4, *p* < 0.05). (**f**) Differentially expressed genes categorized under “Positive regulation of catabolic process” (GO:0009896; Fold change ≥ 2, Volume ≥ 4, *p* < 0.05). (**g**) GO analysis showing “Molecular Function” enrichment among downregulated genes (Fold change ≥ 2, Volume ≥ 4, *p* < 0.05). (**h**) DEGs categorized under “Mitotic nuclear division” (GO:0140014; Fold change ≥ 2, Volume ≥ 4, *p* < 0.05).

To further delineate the mechanisms underlying the phenotypic changes induced by decellularized MRC-5 spheroid scaffolds, transcriptomic profiling was performed on A253 spheroids cultured with or without scaffolds. Heatmaps of DEGs and PCA revealed a clear separation between groups, indicating robust transcriptional reprogramming by the decellularized ECM (Figure 6a,b). Prominent upregulated genes included NEAT1, TXNIP, ATF3, MMP9, TMC4, and PER3 (Figure 6c). Among these, NEAT1, a lncRNA induced under stress, is known to promote the expansion of ALDH1-high populations and confer chemoresistance [37], suggesting a role in the increased ALDH1 expression observed in A/M(DC) spheroids. ATF3 and TXNIP, key regulators of adaptive stress responses, were also elevated. ATF3 functions as a hub of the integrated stress response, supporting tumor survival under metabolic or oxidative stress [38], while TXNIP couples redox regulation with glucose metabolism [39]. These transcriptional shifts are consistent with the enhanced respiratory activity and glucose uptake observed in A/M(DC) cultures. MMP9, a prototypical matrix metalloproteinase, was also upregulated, facilitating ECM degradation and invasion [40], aligning with EMT-like changes and loss of epithelial adhesion observed in Figure 4f. GO analysis further supported these findings, with enriched categories including EGFR and ERBB signaling, proteasomal catabolic activity, PERK-mediated unfolded protein response, integrated stress response, regulation of autophagy, and intrinsic apoptotic signaling (Figure 6d,e). EGFR and ERBB signaling pathways converge on ERK and AKT activation, both of which are well characterized in maintaining stemness and promoting spheroid formation [41]. To validate this mechanism, A/M (DC) spheroids were treated with Erlotinib (EGFR inhibitor) for 24 h. Quantitative RT-PCR showed that ALDH1, CD133, and CK19 expression, elevated in A/M (DC) spheroids, was reduced following EGFR inhibition (Figure S4). These findings confirm that EGFR signaling is a key regulator of the ECM-induced stemness program identified in the transcriptomic analysis. Notably, EGFR-driven signaling has been directly associated with CK19 induction [42], providing a plausible explanation for its increased expression in A/M(DC) spheroids. The enrichment of receptor tyrosine kinase pathways aligns with stromal ligands such as amphiregulin, neuregulin, and HGF identified in MRC-5 spheroids (Figure 2f), likely contributing to the observed proliferation and stemness marker elevation in co-cultures (Figure 1d and Figure 4g). Meanwhile, activation of ER stress-adaptive programs and autophagy is increasingly recognized as a hallmark of chemotherapy resistance [43,44,45], consistent with the cisplatin-resistant phenotype of A/M(DC) spheroids (Figure 5b). Conversely, most downregulated genes were linked to ribosome biogenesis, ribonucleoprotein complex assembly, chromosome segregation, and nuclear division (Figure 6g,h). Suppression of these proliferative and biosynthetic programs, including reduced aurora kinase A (AURKA) and other mitotic regulators, suggests that A/M(DC) spheroids adopt a slower-cycling, stress-adapted state [46]. Such states are characteristic of drug-tolerant persister cells, which resist cytotoxic stress yet retain the ability to re-enter the cell cycle under favorable conditions [47]. In summary, these results demonstrate that decellularized MRC-5 spheroid scaffolds drive A253 cells into a CAF-educated state characterized by receptor tyrosine kinase activation, ECM remodeling, and stress-response pathways, underscoring the value of this simplified yet physiologically relevant stromal platform for modeling tumor–stromal interactions.

## 4. Conclusions

This study establishes a decellularized fibroblast spheroid model that effectively recapitulates tumor–stromal crosstalk in SGC. By preserving ECM architecture and growth factor cues while removing stromal cell interference, this platform not only reproduces key hallmarks of CAF activity such as the induction of cancer stemness, metabolic reprogramming, EMT, and drug resistance, but also enables direct mechanistic validation. Functional inhibition of FAK and EGFR signaling confirmed that these ECM-driven pathways causally mediate the observed stemness and chemoresistant phenotypes. Transcriptomic profiling further revealed that A253 spheroids cultured with decellularized MRC-5 scaffolds adopt a stress-adapted, survival-oriented state resembling in vivo tumor behavior. Collectively, our findings position decellularized fibroblast spheroids as a simplified yet mechanistically informative system for studying tumor progression and evaluating therapeutic responses in SGC.

## Data Availability

The data presented in this study are available on request from the corresponding author. Due to their large file sizes, these datasets cannot be deposited in standard online repositories.

## Supplementary Materials

The following supporting information can be downloaded at: https://www.mdpi.com/article/10.3390/biom15121634/s1, Figure S1: LC-MS full-scan chromatograms of live MRC5 spheroids (Top) and decellularized MRC5 spheroids (Bottom); Figure S2: Validation of ECM-induced proliferation enhancement and stemness in the SGT cell lines; Figure S3: Quantification of E-cadherin fluorescence intensity in A253 spheroids cultured with or without decellularized MRC-5 spheroid scaffolds; Figure S4: Validation of ECM-induced stemness mechanisms by pharmacologic inhibition of FAK and EGFR signaling.

## Abbreviations

The following abbreviations are used in this manuscript:

2D	Two-dimensional
2-NBDG	2-(N-(7-Nitrobenz-2-oxa-1,3-diazol-4-yl) Amino)-2-Deoxyglucose
3D	Three-dimensional
ABC	Ammonium bicarbonate
ALDH1	Aldehyde dehydrogenase 1
ANOVA	Analysis of variance
ATCC	American type culture collection
AURKA	Aurora kinase A
CAF	Cancer-associated fibroblasts
CCK-8	Cell counting kit-8
cDNA	Complementary DNA
CK19	Cytokeratin 19
CLSM	Confocal Laser Scanning Microscope
CSC	Cancer stem cell
DAPI	4′,6-diamidino-2-phenylindole
DEGs	Differentially expressed genes
DMEM	Dulbecco’s Modified Eagle Medium
ECM	Extracellular matrix
EMT	Epithelial–mesenchymal transition
FASP	Filter-Aided Sample Preparation
FBS	Fetal bovine serum
FE-SEM	Field-emission Scanning Electron Microscopy
GAPDH	Glyceraldehyde-3-phosphate dehydrogenase
GO	Gene Ontology
IAA	Iodoacetic acid
IGF	Insulin-like Growth Factor
KEGG	Kyoto Encyclopedia of Genes and Genomes
LC-MS	Liquid Chromatography–Mass Spectrometry
MRC-5	Medical Research Council Cell Strain 5
NCE	Normalized Collisional Energy
NDS	Normal Donkey Serum
NH_3_OH	Ammonium hydroxide
PCA	Principal Component Analysis
PI	Propidium iodide
qRT-PCR	Quantitative real-time polymerase chain reaction
RIPA	Radioimmunoprecipitation assay
SD	Standard deviation
SGC	Salivary gland carcinoma
TCEP	Tris(2-carboxyethyl)phosphine
TME	Tumor microenvironment
ULA	Ultra-low attachment
