# Supplementary material for: Biomimetic Salivary Gland Cancer Spheroid Platform for In Vitro Recapitulation of Three-Dimensional Tumor–Stromal Interactions
Source: Biomolecules. 2025 Nov 21;15(12):1634. doi: 10.3390/biom15121634 (PMC12731055; doi:10.3390/biom15121634)
Supplement: Supplementary file 1 [file biomolecules-15-01634-s001.zip › biomolecules-3905315-supplementary.pdf]

Supplementary Materials

Biomimetic Salivary Gland Cancer Spheroid Platform for in Vitro Recapitulation of Three-dimensional Tumor–stromal Interactions

Lele Wang [1†], Seokjun Kwon [1†], Sujin Park [1], Eun Namkoong [1], Junchul Kim [1], Hye-Young Sim [2,3], Shazid Md. Sharker [4]*and Sang-woo Lee [1]*

[1]Department of Physiology, School of Dentistry and Dental Research Institute, Seoul National University, Seoul 110460, Republic of Korea.

[2]Department of Dentistry and Dental Research Institute, School of Dentistry, Seoul National University, Seoul, 03080, Korea.

[3]Department of Dentistry, SMG-SNU Boramae Medical Center, Seoul, 07061, Korea.

[4]Department of Pharmaceutical Sciences, North South University, Dhaka 1229, Bangladesh.

*Correspondence: Sang-woo Lee, goodman23@snu.ac.kr, Tel. +82-2-740-9766; Shazid Md. Sharker, shazid. sharker@northsouth.edu, Tel. +880-55668200.

†These authors contributed equally to this work.

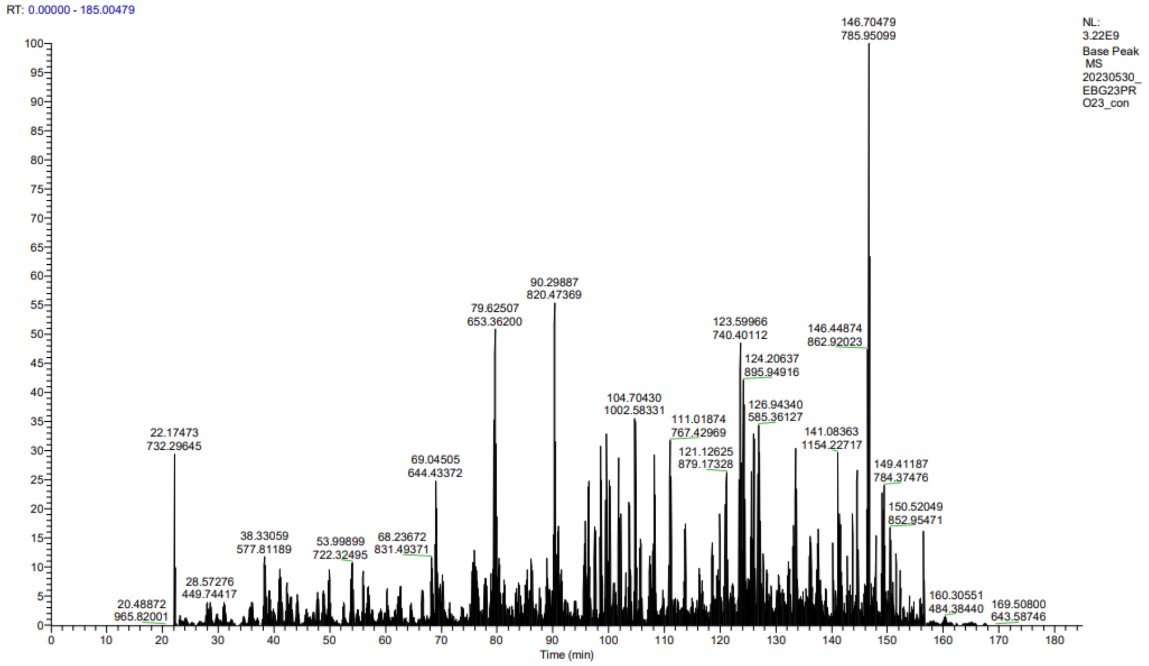

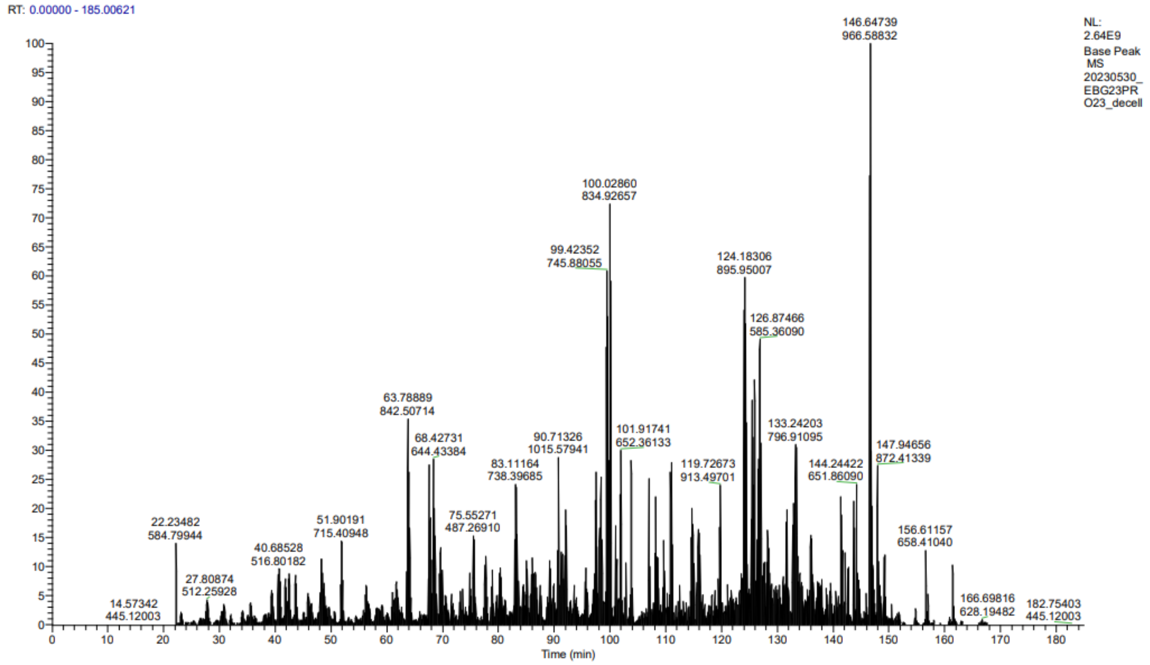

Figure S1. LC-MS full-scan chromatograms of live MRC5 spheroids (Top) and decellularized MRC5 spheroids (Bottom)

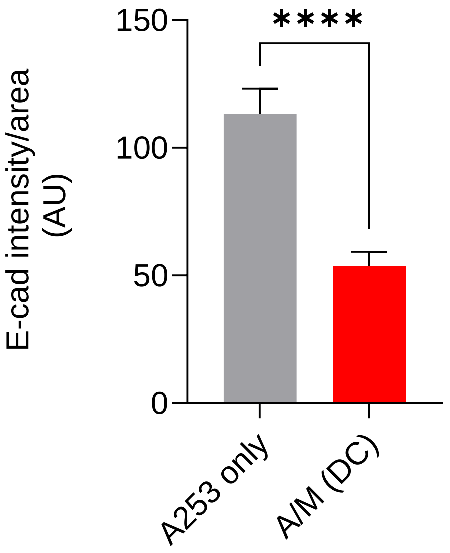

Figure S2. Quantification of E-cadherin fluorescence intensity in A253 spheroids cultured with or without decellularized MRC-5 spheroid scaffolds. Normalized E-cadherin fluorescence intensity per spheroid area in A253-only and A/M (DC) spheroids. Data are presented as mean ± standard deviation (SD, n = 3). Statistical analysis was performed using an unpaired t-test. ****$p < 0.0001$.

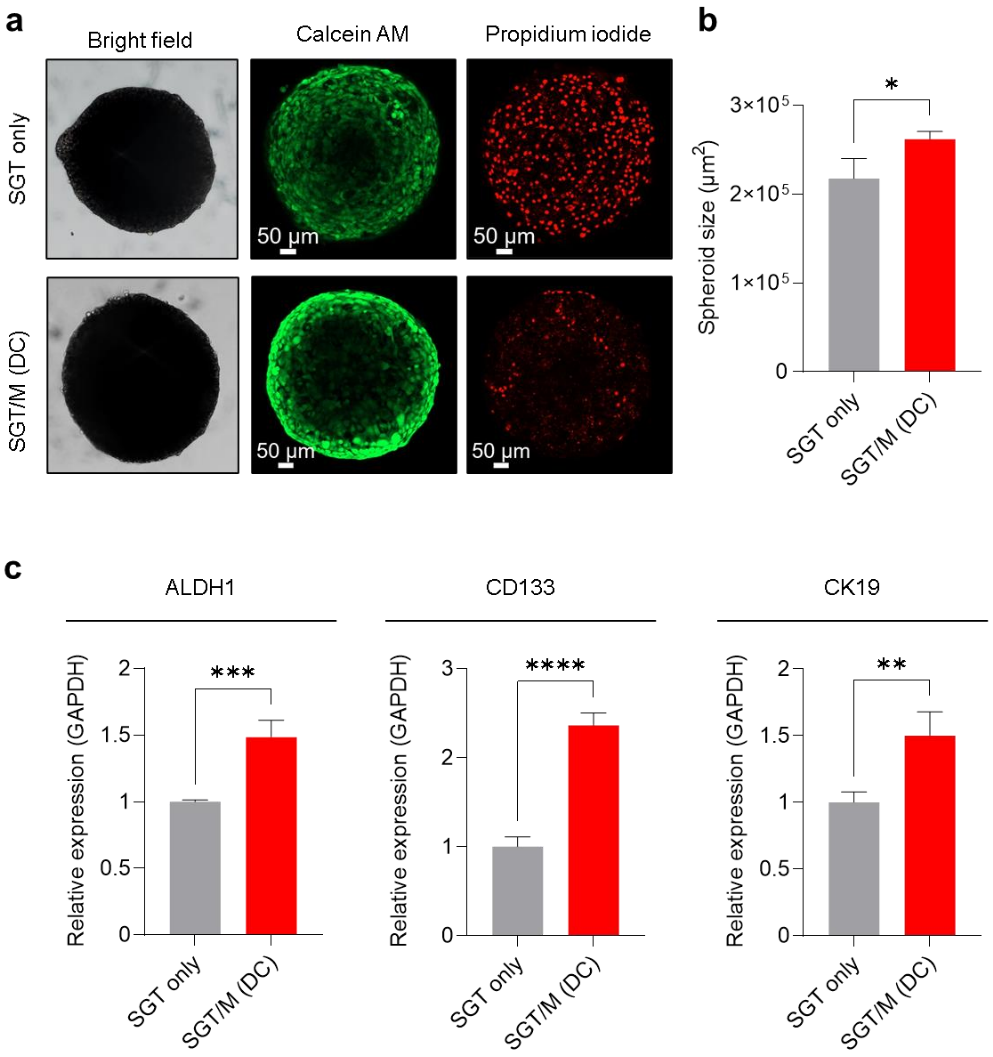

Figure S3. Validation of ECM-induced proliferation enhancement and stemness in the SGT cell lines. (a) Bright-field and Calcein AM/propidium iodide (PI) co-staining images of SGT-only and SGT/M (DC) spheroids. (b) Quantification of spheroid cross-sectional area (μm²) in SGT-only and SGT/M (DC) groups (n = 3). (c) mRNA expression levels of ALDH1, CD133, and CK19 in SGT-only and SGT/M (DC) spheroids analyzed by qRT-PCR (n = 4). Data represent mean ± standard deviation (SD). Statistical analysis was performed using an unpaired t-test. *$p < 0.05$, **$p < 0.01$, ***$p < 0.001$, ****$p < 0.0001$.

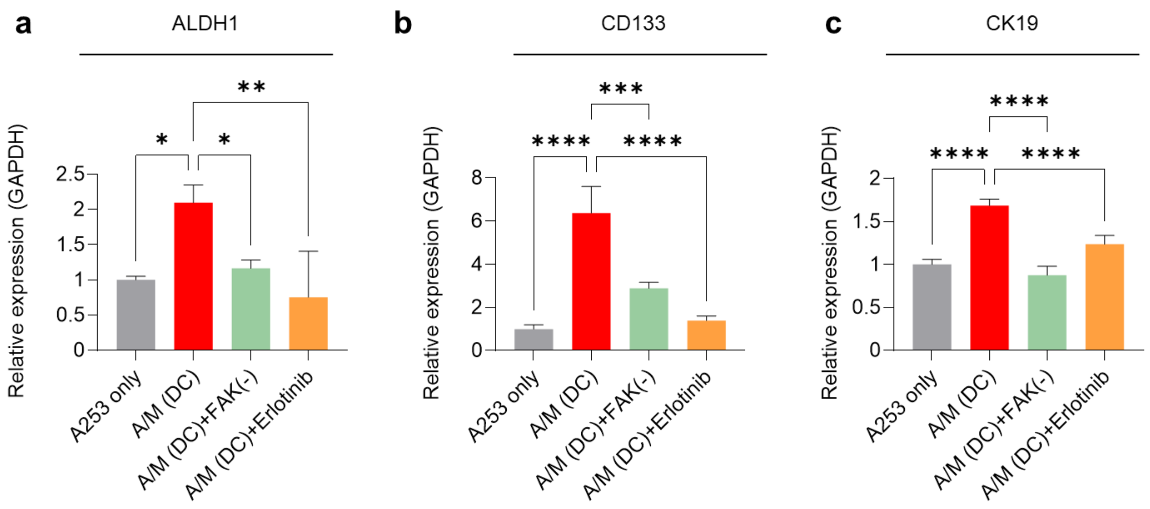

Figure S4. Validation of ECM-induced stemness mechanisms by pharmacologic inhibition of FAK and EGFR signaling. (a) Relative mRNA expression of ALDH1, (b) CD133, and (c) CK19 in A253-only, A/M (DC), A/M (DC)+FAK inhibitor (5 μM, 24 h), and A/M (DC)+Erlotinib (20 μM, 24 h) groups. Expression levels were normalized to GAPDH and are presented relative to A253-only (set to 1). Data represent mean ± SD from three biological replicates. Statistical analysis was performed using one-way ANOVA. *$p < 0.05$, **$p < 0.01$, ***$p < 0.001$, ***$p < 0.0001$.
